# Supplementary material for: Impact of the elderly lung mucosa on Mycobacterium tuberculosis transcriptional adaptation during infection of alveolar epithelial cells
Source: Microbiol Spectr. 2024 Nov 8;12(12):e01790-24. doi: 10.1128/spectrum.01790-24 (PMC11619525; doi:10.1128/spectrum.01790-24)
Supplement: Supplemental material — Fig. S1 to S4; Legend for Tables S1 and S2. [file spectrum.01790-24-s0001.pdf]

Impact of the elderly lung mucosa on *Mycobacterium tuberculosis* transcriptional adaptation during infection of alveolar epithelial cells

SUPPLEMENTARY FIGURES

FIGURE S1

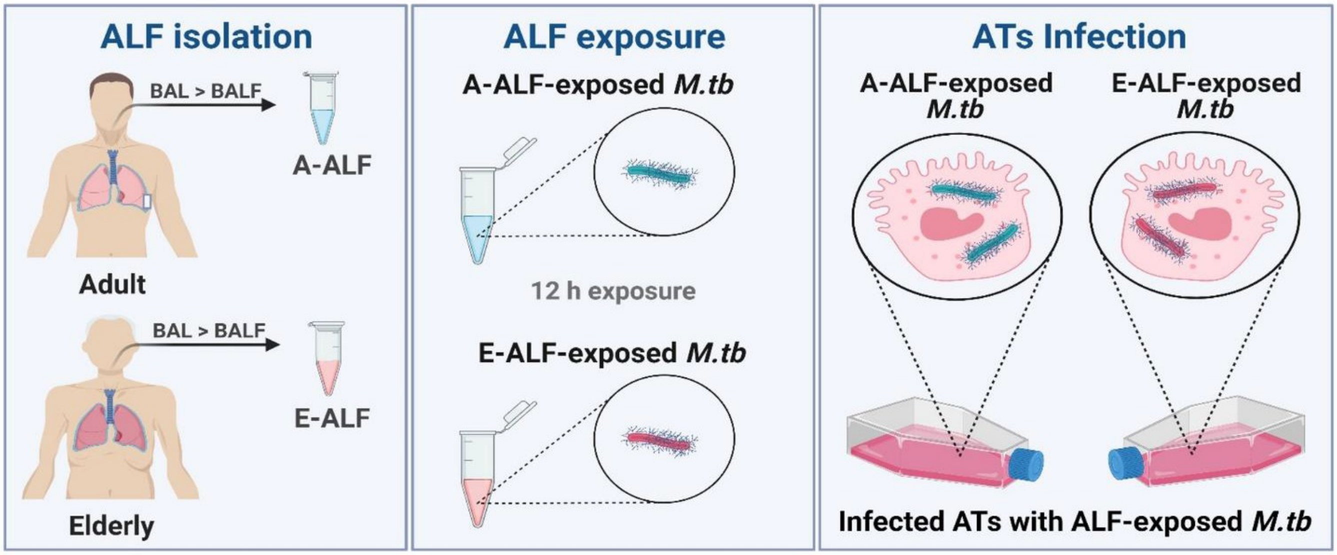

Figure S1. Illustration of the experimental procedures performed in this study. Isolation of ALF from Adult (A-ALF) and Elderly (E-ALF) individuals, followed by A- or E-ALF exposure of *M.tb* and subsequent infection of ATs. Figure created using BioRender (https://biorender.com/).

FIGURE S2

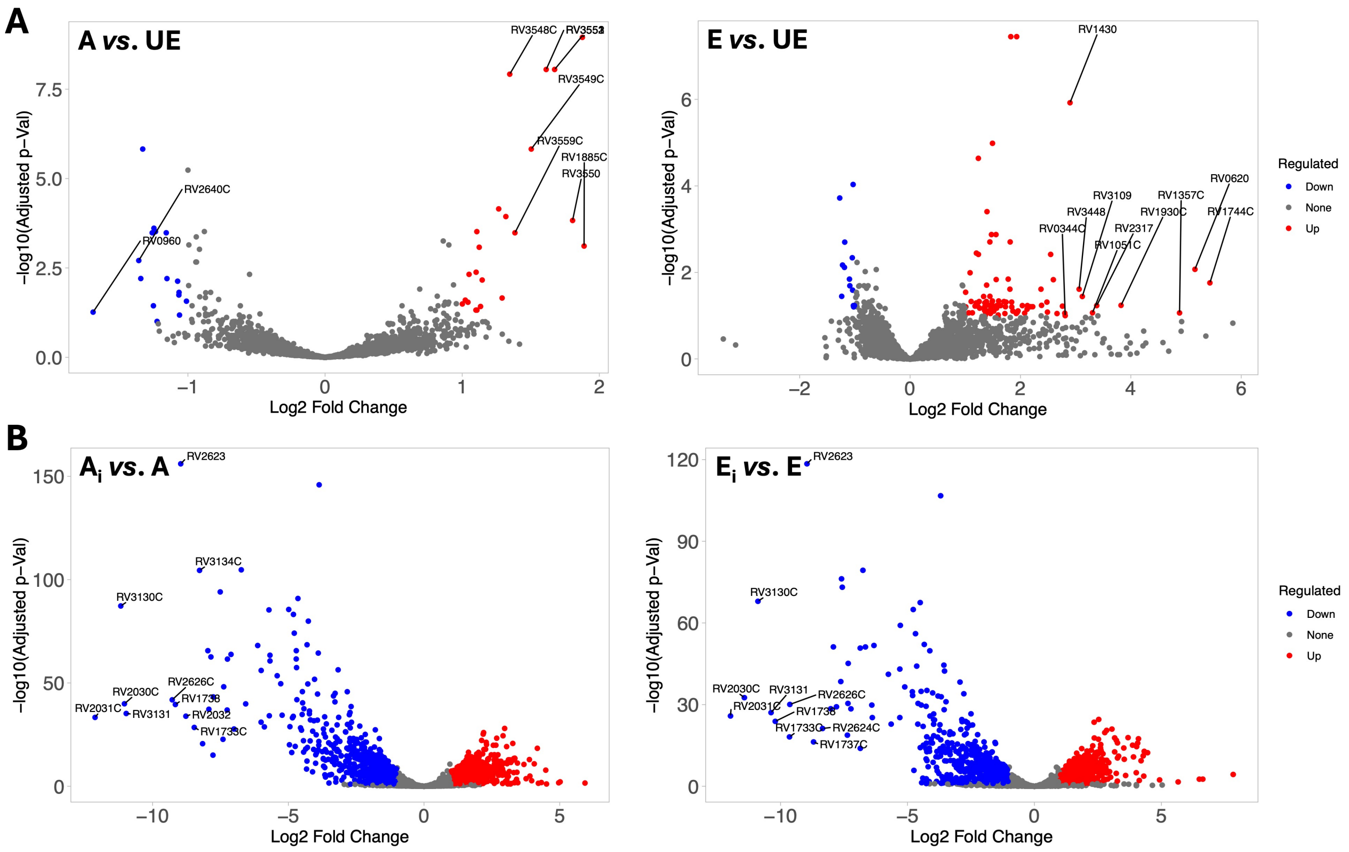

Figure S2. Volcano plots in ALF-exposed *M.tb*. DEGs were used to generate Volcano plots in ALF-exposed *M.tb* prior to (**A**) and during ATs infection (**B**). The top 10 DEGs based on absolute $\log_2$ FC are shown for each comparison. A: A-ALF-exposed *M.tb*; E: E-ALF-exposed *M.tb*; UE: unexposed *M.tb*; A$_i$: A-ALF- exposed *M.tb* during ATs infection; E$_i$: E-ALF-exposed *M.tb* during ATs infection.

FIGURE S3

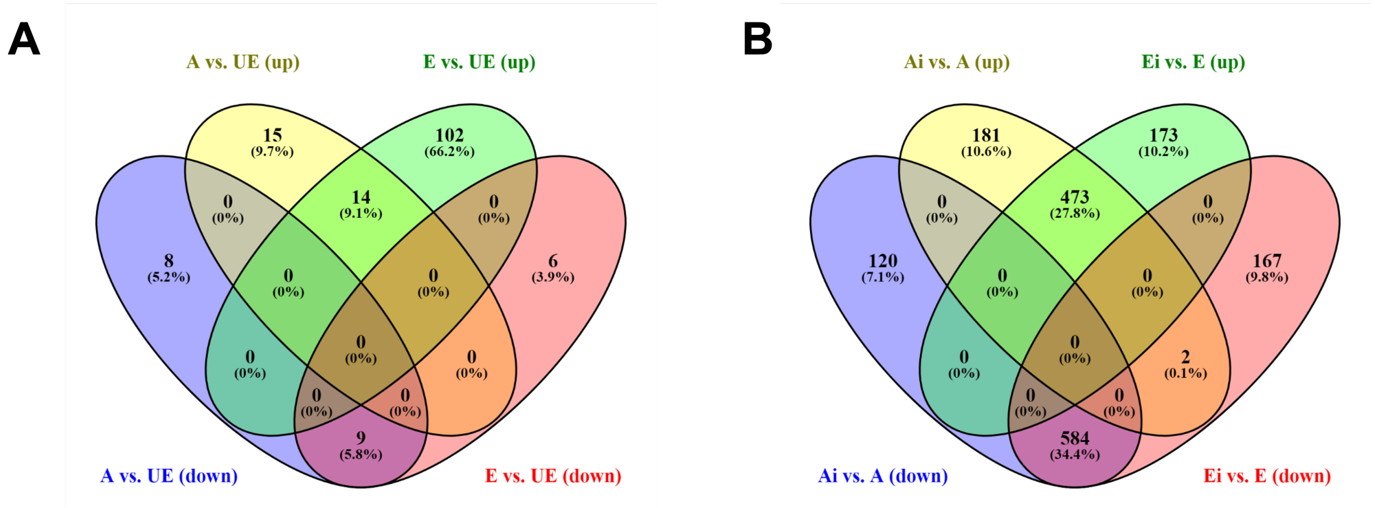

Figure S3. Venn diagram of DEGs in ALF-exposed *M.tb*. Unique and shared DEGs of ALF-exposed *M.tb* prior to (**A**) and during ATs infection (**B**). Venn diagrams were generated using Venny 2.1.0. A: A-ALF-exposed *M.tb*; E: E-ALF-exposed *M.tb*; UE: unexposed *M.tb*; Ai: A-ALF- exposed *M.tb* during ATs infection; Ei: E-ALF-exposed *M.tb* during ATs infection.

FIGURE S4

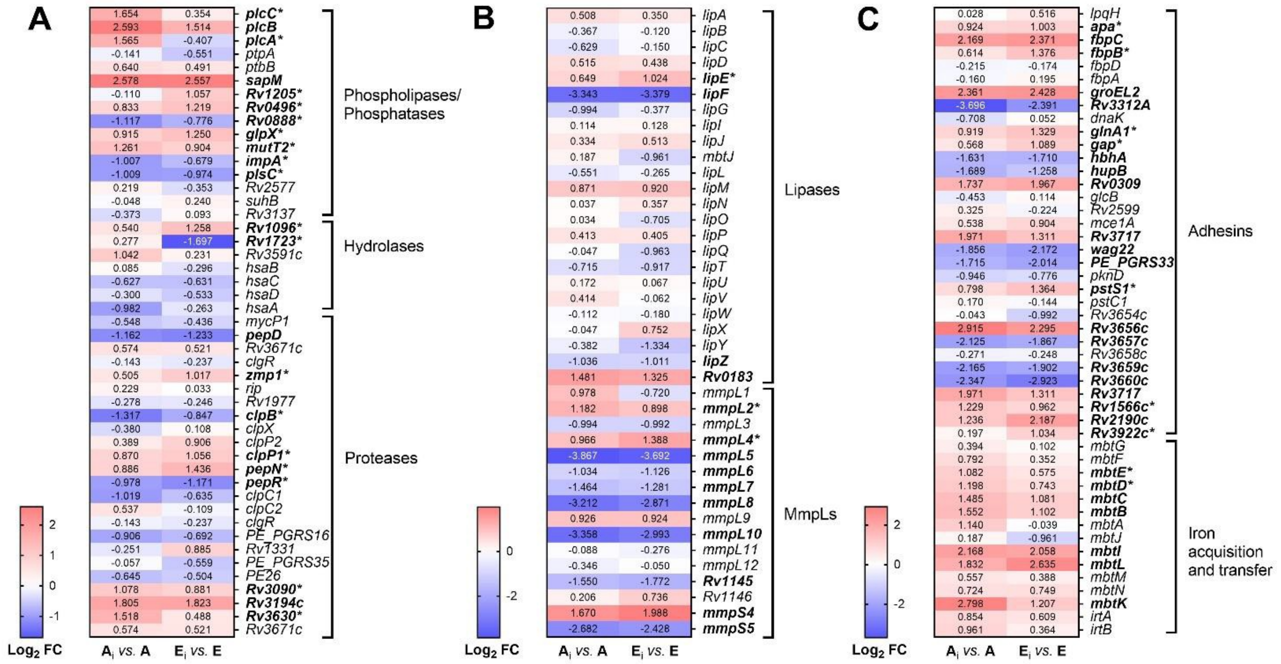

Figure S4. Heatmaps of *M.tb* genes associated with virulence factors at 72 hpi in ATs. (**A**) Phospholipases/Phosphatases, Hydrolases and Proteases; (**B**) Lipases and MmpL; and (**C**) Adhesins during *M.tb* replication in ATs. Cells depict Log2 FC values in ALF-exposed *M.tb* in ATs (A_i: Adult ALF-exposed *M.tb* during infection; E_i: Elderly ALF-exposed *M.tb* during infection) *vs*. ALF- exposed *M.tb* before infection (A: Adult ALF; E: Elderly ALF), upregulated: red, downregulated: blue. Genes in bold indicate significant DEGs (Log2 FC equal or greater than an absolute value of 1, FDR < 0.1) for both conditions. Genes with an asterisk indicate a significance in only one of the comparisons, highlighting differences between conditions. Note the different scales used in A, B, and C for better visualization of the results. Heatmaps were generated in GraphPad Prism v9.1.1.

SUPPLEMENTARY TABLES

Table S1. Complete list of *M.tb* DEGs (upregulated and downregulated) after exposure to ALF before and during ATs infection at 72 hpi. DE is shown as Log2 FC; up- and downregulated genes in orange and blue, respectively; significant DEGs (Log2 FC equal or greater than an absolute value of 1 and FDR < 0.1) are highlighted in bold. DEGs are listed based on locus tag, and a description of functional category is included (Mycobrowser and Uniprot).

Table S2. DE analysis of *M.tb* genes (all genes) after exposure to ALF before and during ATs infection at 72 hpi. DE is shown as Log2 FC; significant DEGs (Log2 FC equal or greater than an absolute value of 1 and FDR < 0.1) are highlighted in bold. DEGs are listed based on locus tag, and a description of functional category is included (Mycobrowser and Uniprot).
